# Seungmagalgeun-Tang, a Traditional Herbal Formula, Alleviates Skin Inflammation and Depression-Like Behavior in Atopic Dermatitis Mice under Sleep Deprivation Conditions

**DOI:** 10.1155/2022/1307173

**Published:** 2022-03-23

**Authors:** Ly Thi Huong Nguyen, Min-Jin Choi, Heung-Mook Shin, In-Jun Yang

**Affiliations:** ^1^Department of Physiology, College of Korean Medicine, Dongguk University, Gyeongju 38066, Republic of Korea; ^2^R&D Center, Etnova Therapeutics Corp., 124, Sagimakgol-ro, Jungwon-gu, Gyeonggi-do 13207, Republic of Korea

## Abstract

Atopic dermatitis (AD) is a common inflammatory skin disease, which can be worsened under sleep deprivation (SD) conditions. This study investigated the efficacy and the mechanism of action of the traditional herbal formula Seungmagalgeun-tang (SMGGT) on the inflammation and behavioral changes in a mouse model of AD exposed to SD. SMGGT decreased levels of IgE, TNF-*α*, IL-4, IL-13, and mast cell infiltration and reduced the expression of CD3 in the mouse skin. SMGGT also reversed the SD-induced increase in corticosterone and decrease in melatonin level. Furthermore, SMGGT reduced the immobility time in the tail suspension test significantly. HaCaT cells and HMC-1 cells were used to investigate the effects of SMGGT on cell signaling pathways. In TNF-*α*/IFN-*γ* (TI) treated HaCaT cells, SMGGT reduced production of TARC/CCL17 and MDC/CCL22 and suppressed the p38 MAPK, STAT1, and NF-*κ*B pathways. In substance P (SP)/CRH-stimulated HMC-1 cells, SMGGT decreased VEGF production and inhibited ERK phosphorylation. Network pharmacology and molecular docking analysis revealed that puerarin and paeoniflorin might contribute to the effects of SMGGT by targeting several AD-related molecules and pathways. Puerarin and paeoniflorin exerted anti-inflammatory effects by decreasing production of MDC/CCL22 and IL-6 in TI-treated HaCaT cells and VEGF production in SP/CRH-stimulated HMC-1 cells. This study suggests that SMGGT with puerarin and paeoniflorin as main bioactive components alleviates skin inflammation and depression-like behavior in a sleep-deprived mouse model of AD.

## 1. Introduction

Up to 87% of adults and 80% of children with atopic dermatitis (AD) suffer from sleep deprivation (SD). Nocturnal itching and scratching movements can severely disrupt the continuity and quality of sleep in AD patients [1]. Poor sleep at night can trigger an awareness of itching, leading to more scratching by these patients, indicating a vicious cycle between AD and SD [2]. A recent clinical trial reported the efficacy of melatonin supplementation on both sleep quality and skin symptoms in pediatric patients with AD [3]. Hence, the management of SD might be a promising approach for the treatment of AD.

The cellular and molecular mechanisms underlying SD that could worsen AD have been suggested [1]. Acute and chronic SD can promote inflammation by increasing the production of various inflammatory cytokines, including TNF-*α*, IL-1*β*, IL-6, and IL-17 [4]. SD has also been reported to disrupt the normal functions of regulatory T cells and alter the Th1/Th2 balance toward Th2 dominance [5]. AD patients with sleep loss show a decrease in the ratio between the level of Th1 cytokine IFN-*γ* and Th2 cytokine IL-4 than those without AD [6]. Moreover, SD could trigger psychological stress in AD patients, leading to a high risk of anxiety and depression [1]. SD-induced stress activates the hypothalamic-pituitary-adrenal (HPA) axis to stimulate the secretion of stress hormones, including corticotropin-releasing hormone (CRH) produced by the hypothalamus, adrenocorticotropic hormone (ACTH) from the pituitary gland, and glucocorticoids (cortisol in human or corticosterone in mouse/rat, CORT) from the adrenal gland [7]. Eight days of SD significantly increased CORT plasma level in a murine model of autoimmune disease [8]. CORT can disrupt skin barrier function and shift the immunity toward the Th2 response [9, 10]. CRH, another stress hormone, can activate mast cells in the skin and increase vascular permeability [11]. Activated mast cells can secret histamine and a range of inflammatory molecules, aggravating the itching symptoms in AD [12].

Seungmagalgeun-tang (SMGGT) is a traditional herbal prescription that consists of four medicinal herbs: Puerariae Radix, Glycyrrhizae Radix, Paeoniae Radix, and Cimicifugae Rhizoma. According to “*Collection of Prescriptions with Notes* (Yifang Jijie),” a classic book of traditional medicine, SMGGT has been used to treat both skin diseases, including purple rash, urticarial, and sleep disturbance. Previous studies reported that SMGGT had anti-inflammatory effects by reducing the expression of proinflammatory cytokines IL-4, IL-6, IL-8, and TNF-*α* in mast cells [13, 14]. Paeoniae Radix, a herbal constituent of SMGGT, was reported to improve sleep quality in patients with restless leg syndrome [15]. However, the therapeutic capacity of SMGGT on AD exaggerated by SD has not been elucidated. Hence, the current study investigated the effects and mechanism of action of SMGGT extract on skin inflammation and behavioral changes in a mouse model of AD under SD conditions. In addition, network pharmacological analysis was performed to investigate the bioactive components of SMGGT and their possible targets for the treatment of AD.

## 2. Materials and Methods

### 2.1. Preparation of Seungmagalgeun-Tang (SMGGT)

SMGGT was obtained from Kyung Hee University Hospital (Seoul, Korea). SMGGT (Puerariae Radix, Glycyrrhizae Radix, Cimicifugae Rhizoma, and Paeoniae Radix; weight ratio 1 : 1 : 1 : 1) (Table 1) was extracted using 30% ethanol for 3 h at 70°C. The crude extract was filtered using filter papers (pore size: 8 *μ*m, Whatman International, Maidstone, UK), concentrated by using a rotary evaporator (EYELA N-1100, Tokyo Rikakikai, Tokyo, Japan), and lyophilized by using a freeze-drying machine (Ilshin, Busan, Korea). The dried SMGGT (yield 24.9% w/w) obtained was dissolved in DMSO, passed through a 0.22 *μ*m syringe filter (Sartorius, Goettingen, Germany), and stored at −20°C.

### 2.2. High-Performance Liquid Chromatography (HPLC) Analysis

Albiflorin, paeoniflorin, liquiritigenin, glycyrrhizic acid, ferulic acid, puerarin, and daidzin were obtained from ChemFaces (Wuhan, China). Analysis of chemical constituents was conducted using an HPLC 1290 system (Agilent, Santa Clara, CA, USA). The SMGGT extract and standard samples were separated on a YMC-Triart C18 column (2.1 × 100 mm, 5 *μ*m; YMC America, PA, USA) with two mobile phases: (A) 0.1% phosphoric acid and (B) acetonitrile. The solvent gradient was 10–20% (B) for 6 min and 20–65% (B) for 9 min, followed by 5 min equilibration. The column temperature was set at 30°C, and the flow rate was 0.3 ml/min. Albiflorin, liquiritigenin, and paeoniflorin were detected at 230 nm wavelength. Daidzin, glycyrrhizic acid, and puerarin were detected at 250 nm wavelength. Ferulic acid was detected at 320 nm wavelength. The concentrations of the compounds in SMGGT were calculated using calibration lines.

### 2.3. Animal Model and Treatments

**Figure 1:**
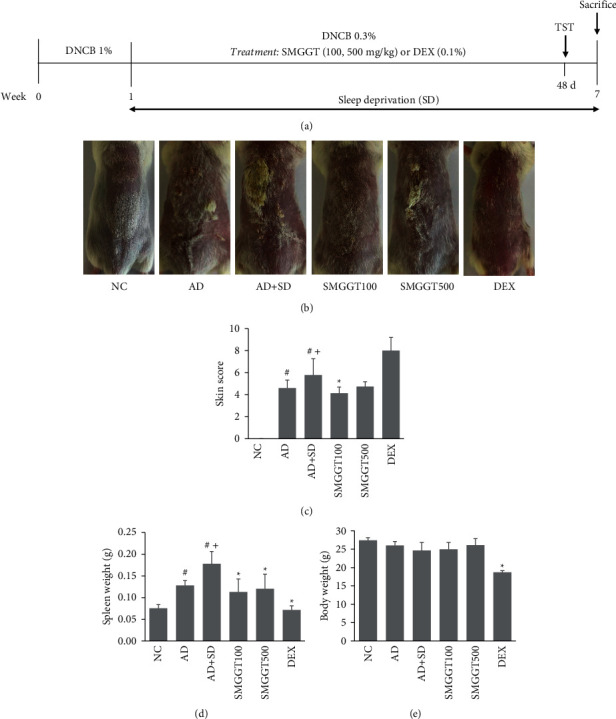
SMGGT alleviated skin symptoms in AD mice under SD conditions. (a) Experimental schedule. (b) Representative photos of mice from six groups. Skin severity scores (c), spleen weights (d), and body weights (e) were recorded. Data are presented as means ± SDs (*n* = 5 per experiment). ^#^*P* < 0.05 vs. the NC group; ^+^*P* < 0.05 vs. the AD group; ^*∗*^*P* < 0.05 vs. the AD + SD group.

Male BALB/c mice (five weeks old, *n* = 30) were procured from Koatech Laboratory Animal Inc. (Seoul, Korea). All animal experiments were conducted following the protocols from the Institutional Animal Care and Use Committee of Dongguk University (IACUC-2019-7). The animals were stabilized for seven days before starting the experiments. The mice were assigned randomly to six groups: normal control group (the NC group), DNCB treatment group (the AD group), DNCB + sleep deprivation (SD) group (the AD + SD group), DNCB + SD + 100 mg/kg of SMGGT (the SMGGT100 group), DNCB + SD + 500 mg/kg of SMGGT (the SMGGT500 group), and DNCB + SD + 0.1% dexamethasone (the DEX group).  Figure 1(a) presents the experimental design diagram. DNCB and DEX for this experiment were obtained from Sigma-Aldrich (St. Louis, MO, USA). The dorsal skin area of each mouse was shaved, and then 200 *μ*l of 1% DNCB (soluble in acetone/olive oil solution, 3 : 1 v/v) was treated three times for one week (sensitization). In the challenge period, 0.3% DNCB (200 *μ*l) was treated to the mouse skins three times a week for six weeks. The mice were orally administered 100 or 500 mg/kg of SMGGT or topically treated with 0.1% DEX every day for six weeks. The SMGGT doses used in this experiment were the doses of other traditional herbal formulas used to treat AD-like symptoms in mouse models in previous studies [16, 17]. There were no side effects, abnormal behavior, or weight loss when the highest concentration of SMGGT was administered. Sleep deprivation was induced as described previously with modifications [18]. The mice were allowed to run on a treadmill at a slow speed (10 m/min) during the day (four cycles of 1 h running and 1 h resting, between 10 : 00 and 18 : 00). After running on the treadmill, the mice were put back in their normal cages. On the last day of the experiment, the skin severity score was recorded, including four different skin symptoms: redness, dryness, edema, and excoriation, with four intensity levels: none (0), mild (1), moderate (2), and severe (3). For sacrifice, the mice were anesthetized using isoflurane and the body weights were evaluated. The spleens were dissected, and the spleen weights were recorded. The skin and blood samples were collected for further analysis.

### 2.4. Tail Suspension Test (TST)

To assess depression-like behavior in mice, the TST was performed, as described previously [19, 20]. The mice were acclimated to the test room for 2 h before testing. The mice were hung on a bar by a 15 cm-long adhesive tape attached approximately 1 cm from the tail tip. The distance from the head of the mouse to the floor was set at 50 cm. Each test session was conducted for 6 min. The immobility time was evaluated as the time without any limb movements.

### 2.5. Histological and Immunohistochemical Analysis

The skin tissues were fixed using 4% paraformaldehyde and then embedded into paraffin. Skin sections were prepared at a thickness of 5 *μ*m, and then hematoxylin and eosin (H&E) and toluidine blue (TB) staining of these sections were performed. For immunohistochemical staining, the sections were incubated with the anti-CD3 antibody (1 : 100, #ab5690, Abcam, Cambridge, MA) at 4°C overnight. The samples were then incubated with the anti-rabbit horseradish peroxidase (HRP)-conjugated secondary antibody for 1 h at room temperature. All stained samples were visualized using a Lionheart FX automated microscope and Gen5 software (BioTek Instruments, Winooski, VT, USA). The number of mast cells infiltrated in the dermis layer was counted. The CD3 intensity was evaluated by ImageJ Fiji software (WS Rasband, National Institutes of Health, Bethesda, MD, USA).

### 2.6. Cell Culture and Treatments

A human immortalized keratinocyte cell line (HaCaT cells) was provided by Korea Institute of Oriental Medicine (Daegu, Korea) and maintained in high glucose DMEM medium (Welgene Inc., Gyeongsangbuk, Korea) supplemented with 10% FBS and 1% antibiotics (10,000 *μ*g/ml of streptomycin and 10,000 units/ml of penicillin) (Invitrogen Inc., Carlsbad, CA, USA) and incubated at 37°C with 5% CO_2_. After serum-starvation for 24 h, HaCaT cells were pretreated with SMGGT (10 and 100 *μ*g/ml), puerarin, paeoniflorin (1, 10 *μ*M), or DEX (10 *μ*M) for 1 h and then incubated with TNF-*α* and IFN-*γ* (TI; 10 ng/ml each) for the indicated times. Recombinant human TNF-*α* and IFN-*γ* were obtained from Koma Biotech Inc. (Seoul, Korea).

A human mast cell line (HMC-1 cells) was purchased from Merck Millipore (Darmstadt, Germany) and grown in Iscove's Modified Dulbecco's Medium (IMDM) (Merck Millipore) supplemented with 10% FBS, 1% antibiotics, and 1.2 mM 1-thioglycerol (Sigma-Aldrich, St. Louis, MO, USA), at 37°C with 5% CO_2_. The cells were stimulated with 10 *μ*M SP for 48 h, followed with 1 nM CRH for 24 h, as described previously [21]; SMGGT (10 and 100 *μ*g/ml), puerarin, paeoniflorin (1, 10 *μ*M), or DEX (10 *μ*M) was treated 1 h before addition of CRH. SP and CRH were obtained from Sigma-Aldrich (St. Louis, MO, USA).

### 2.7. Cell Viability

The effects of SMGGT on cell viability were determined using XTT assays. HaCaT and HMC-1 cells were treated with SMGGT (10, 100, 500, and 1000 *μ*g/ml) or puerarin and paeoniflorin (1 and 10 *μ*M) for 24 h and then incubated with 50 *μ*l of XTT solution (Sigma-Aldrich, St. Louis, MO, USA) for 4 h. The absorbance was evaluated using a microplate reader (Tecan, Männedorf, Switzerland) at 450 nm wavelength (reference wavelength at 650 nm). The cell viability was presented as a percentage of the untreated controls.

### 2.8. Western Blotting

Dorsal skin samples were homogenized using a tissue extraction reagent with protease and phosphatase inhibitors (Thermo Fisher Scientific, Vienna, Austria). The skin lysates were centrifuged at 10,000 × *g* for 20 min at 4°C, and the supernatants were then collected. HaCaT cells were harvested and lysed in RIPA lysis buffer (150 mM NaCl, 20 mM HEPES, 0.5% sodium deoxycholate, 0.1% SDS, 1.0% IGEPAL CA-630, pH 7.5) containing a cocktail of protease and phosphatase inhibitors (Atto, Tokyo). The supernatants were obtained by centrifugation at 8000 × *g* for 15 min at 4°C. The protein concentrations were evaluated using Bradford assays. Protein samples (25–50 *μ*g) were separated by SDS-PAGE electrophoresis and then transferred onto the PVDF membranes (Merck Millipore, Darmstadt, Germany). The membranes were then blocked using a 5% skim milk solution for 2 h. After washing with 1X PBS, the membranes were incubated with the primary antibodies at 4°C overnight and then with the HRP-conjugated anti-IgG secondary antibodies. Primary antibodies for phospho-p38 (#9211, 1 : 2500), phospho-JNK (#9251, 1 : 1500), phospho-ERK (#4370, 1 : 2500), phospho-STAT1 (#9167, 1 : 2500), p38 (#9212, 1 : 2500), ERK (#9102, 1 : 2500), NF-*κ*B (#8242, 1 : 2500), and I*κ*B-*α* (#4814, 1 : 2500) were procured from Cell Signaling Technology (Danvers, MA, USA). The antibodies for JNK (sc-474, 1 : 2500) and phospho-I*κ*B-*α* (sc-101713, 1 : 1500) were obtained from Santa Cruz (Dallas, TX, USA). Anti-*β*-actin antibody (A1978, 1 : 10,000) was acquired from Sigma-Aldrich (St. Louis, MO, USA). The antibody against lamin B2 (ab151735, 1 : 2500) was supplied by Abcam (Cambridge, MA, USA). The secondary antibodies, including HRP-conjugated goat anti-rabbit IgG (#31460, 1 : 5000) and goat anti-mouse IgG (#32430, 1 : 5000), were obtained from Invitrogen Inc. (Carlsbad, CA, USA). The blots were visualized using enhanced chemiluminescence substrates and the ChemiDoc imaging system (Bio-Rad, Hercules, CA, USA). The intensities of the protein bands were calculated using Gel-Pro V3.1 (Media Cybernetics, MD, USA).

### 2.9. Enzyme-Linked Immunosorbent Assay (ELISA)

The skin samples were homogenized and centrifuged as mentioned before. The supernatants were collected, and protein samples were prepared at a concentration of 0.5 mg/ml. The levels of cytokines and chemokines in skin lysates, serum, and cell culture media were assessed using commercial ELISA kits according to protocols from manufacturers. Human TARC/CCL17 and MDC/CCL22 and mouse IL-4 and IL-10 ELISA kits were purchased from R&D Systems (Minneapolis, MN, USA). Human IL-6 and VEGF and mouse IgE, VEGF, IL-13, and TNF-*α* ELISA kits were obtained from Koma Biotech Inc. (Seoul, Korea). Mouse CORT ELISA kits were supplied by Arigo Biolaboratories (Hsinchu, Taiwan). Mouse melatonin kits were acquired from LifeSpan BioSciences (Seattle, WA, USA). The absorbances were determined using a microplate reader (Tecan, Männedorf, Switzerland) at wavelengths of 450–550 nm.

### 2.10. Network Pharmacological Analysis

Network pharmacological analysis was performed to investigate the targets of verified compounds of SMGGT and their related signaling pathways. The Traditional Chinese Medicine Systems Pharmacology (TCMSP) database [22] was used to predict target genes of the components of SMGGT. The compound-target network was visualized using Cytoscape 3.8.2 (Cytoscape Consortium, CA, USA). Cytoscape DisGeNET App was used to access atopic dermatitis-related genes [23]. Kyoto Encyclopedia Genes and Genomes (KEGG) and Gene Ontology (GO) enrichment analysis for biological processes were conducted using the Enrichr tool (https://maayanlab.cloud/Enrichr/) [24].

### 2.11. Molecular Docking Analysis

Binding between SMGGT compounds and target protein was estimated using AutoDock Vina software (https://vina.scripps.edu/download.html) [25]. For docking analysis, the 3D structures of IFN-*α*, TNF-*α*, and COX-2 with protein data bank (PDB) format were downloaded from the RCSB protein database (https://www.rcsb.org/) and 3D structures of SMGGT compounds were acquired from PubChem database (https://pubchem.ncbi.nlm.nih.gov/). Binding affinities between compounds and targets were investigated using AutoDock tools and visualized using PyMOL 2.5 program (https://pymol.org/2/) [26].

### 2.12. Statistical Analysis

Statistical analysis was performed using GraphPad Prism 5 software (GraphPad Software, CA, USA). To examine the difference between the two groups, Student's *t*-test for unpaired experiments was performed. All experiments in the present study were conducted at least three times independently. Data are presented as the mean ± standard deviation (SD). Pearson correlation was used to evaluate the correlation between two different parameters. A level of *P* < 0.05 was considered statistically significant.

## 3. Results

### 3.1. SMGGT Alleviated Skin Symptoms in AD Mice under SD Conditions

SD exacerbated the AD symptoms significantly in the DNCB-treated mice. In contrast, treatment with SMGGT at 100 mg/kg reduced the SD-induced increases in the severity of skin symptoms (Figures 1(b) and 1(c)). The AD + SD group showed higher spleen weights compared with the AD group, and SMGGT at 100 and 500 mg/kg reduced these increases (Figure 1(d)). The body weights of SMGGT-treated groups were not significantly different from the NC group (Figure 1(e)).

### 3.2. SMGGT Reduced Inflammation in AD Mice under SD Conditions

**Figure 2:**
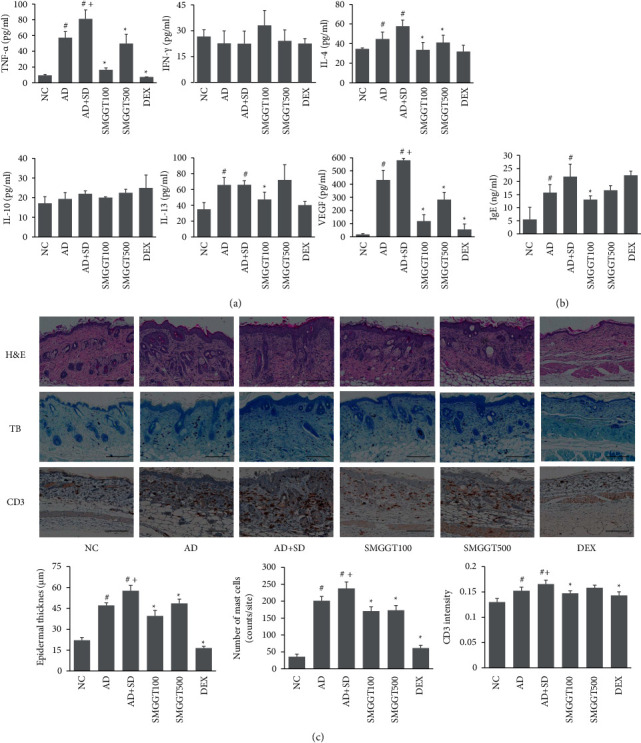
SMGGT reduced inflammation in AD mice under SD conditions. Levels of inflammatory cytokines in skin lysates (a) and serum IgE levels (b) were evaluated. (c) Histological and immunohistochemical staining of skin samples. Scale bar: 200 *μ*m. Data are presented as the means ± SDs (*n* = 5 per experiment). ^#^*P* < 0.05 vs. the NC group; ^+^*P* < 0.05 vs. the AD group; ^*∗*^*P* < 0.05 vs. the AD + SD group.

The AD + SD group showed higher skin levels of inflammatory cytokines, including IL-4, IL-13, TNF-*α*, and VEGF, compared with the NC group. Interestingly, TNF-*α* and VEGF production in the AD + SD group was significantly higher than that in the AD group. In contrast, treatment with SMGGT (100 and 500 mg/kg) significantly decreased the expression of TNF-*α*, IL-4, and VEGF in skin lysates, and the SMGGT100 group showed lower levels of IL-13 than the AD + SD group (Figure 2(a)). Moreover, serum IgE levels in the AD and AD + SD groups were significantly higher than those in the NC group, but these increases were reduced by treatment with SMGGT (100 mg/kg) (Figure 2(b)).

The AD + SD mice had a thicker *epidermis* layer and a higher number of mast cell infiltrations than the AD mice (Figure 2(c)). However, SMGGT treatment (100 and 500 mg/kg) significantly decreased the epidermal thickness and the number of mast cells compared with the AD + SD group (Figure 2(c)). In addition, the AD + SD group had a higher CD3 intensity than the AD group, which was reduced by treatment with SMGGT at 100 mg/kg (Figure 2(c)).

### 3.3. SMGGT Improved the Behavioral Change and Hormone Levels in AD Mice under SD Conditions

**Figure 3:**
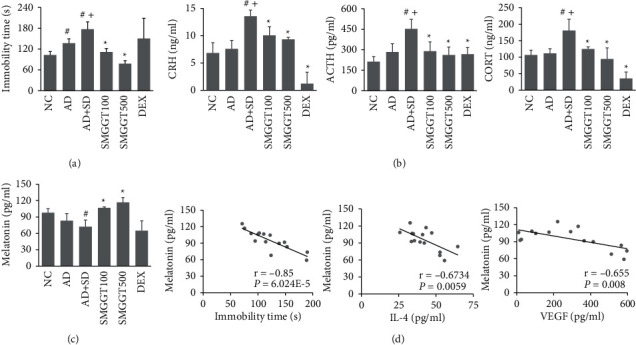
SMGGT improved the behavioral change and hormone levels in AD mice under SD conditions. (a) Immobility time in the TST was measured. (b) Melatonin levels in serum. (c) CRH, ACTH, and CORT levels in serum. (d) Pearson correlation between the serum melatonin levels and immobility time in the TST and skin IL-4 and VEGF levels. Data are presented as the means ± SDs (*n* = 5 per experiment). ^#^*P* < 0.05 vs. the NC group; ^+^*P* < 0.05 vs. the AD group; ^*∗*^*P* < 0.05 vs. the AD + SD group.

SD was suggested to promote depression-like behavior in a mouse model [27]. In this study, the AD + SD mice increased the immobility time in the TST significantly, in comparison to the NC and AD groups. On the other hand, this increase was suppressed significantly by treatment with SMGGT (100 and 500 mg/kg) (Figure 3(a)). The levels of stress-related hormones, including CRH, ACTH, and CORT, were higher in the AD + SD group than in the NC and the AD group, which were decreased significantly in the SMGGT treatment groups (Figure 3(b)). The serum levels of melatonin were also lower in the AD + SD group than in the NC group, which recovered with SMGGT administration (Figure 3(c)). Moreover, the melatonin levels had negative correlations with the immobility time in the TST and the levels of inflammatory cytokines IL-4 and VEGF in skin lesions (Figure 3(d)).

### 3.4. SMGGT Reduced TI-Induced Inflammation in HaCaT Cells

**Figure 4:**
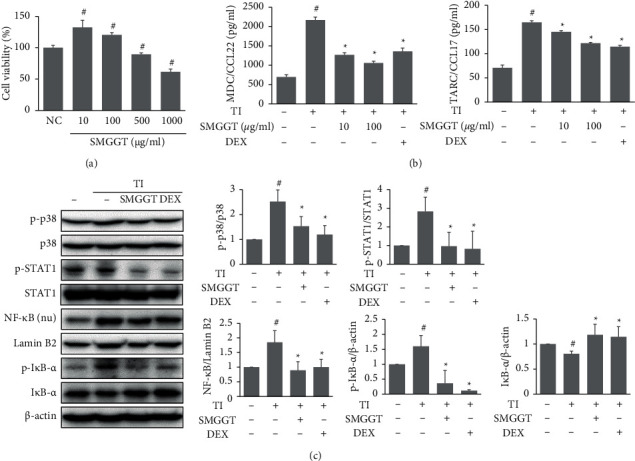
SMGGT reduced TI-induced inflammation in HaCaT cells. (a) The viability of HaCaT cells was evaluated using XTT assays. (b) TARC/CCL17 and MDC/CCL22 levels in cell culture supernatants. (c) Protein levels of phosphorylated form and total form of p38, STAT1, I*κ*B-*α*, and NF-*κ*B in cell lysates. Data are presented as the means ± SDs (*n* = 3 per experiment). ^#^*P* < 0.05 vs. untreated control cells (NC); ^*∗*^*P* < 0.05 vs. TI-treated cells.

To understand the action mechanism of the SMGGT, this study examined its effects in HaCaT cells. The effects of SMGGT on the viability of HaCaT cells were measured by XTT assays, and following experiments were conducted at nontoxic concentrations (Figure 4(a)). The levels of Th2-attracting chemokines (TARC/CCL17 and MDC/CCL22) were increased in HaCaT cells upon TI stimuli, but SMGGT (10 and 100 *μ*g/ml) reduced these increases significantly (Figure 4(b)). Furthermore, SMGGT (100 *μ*g/ml) significantly suppressed TI-induced phosphorylation of p38 and STAT1, as well as nuclear translocation of NF-*κ*B p65 by downregulating phosphorylation of I*κ*B-*α* (Figure 4(c)).

### 3.5. SMGGT Decreased SP/CRH-Induced Inflammation in HMC-1 Cells

**Figure 5:**
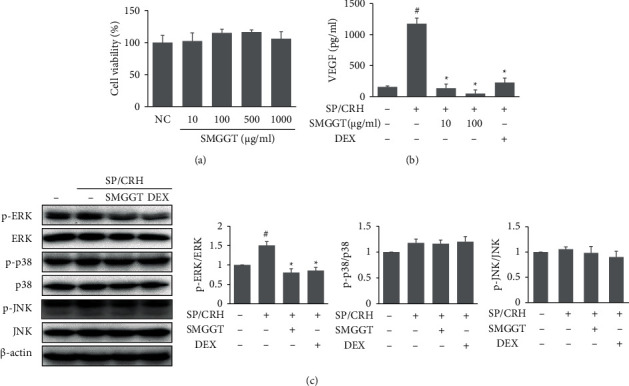
SMGGT decreased SP/CRH-induced inflammation in HMC-1 cells. (a) The viability of HMC-1 cells was evaluated using XTT assays. (b) VEGF level in cell culture supernatants. (c) Protein levels of phosphorylated form and total form of ERK, p38, and JNK in cell lysates. Data are presented as means ± SDs (*n* = 3 per experiment). ^#^*P* < 0.05 vs. untreated control cells (NC); ^*∗*^*P* < 0.05 vs. SP/CRH-treated cells.

SP/CRH-treated HMC-1 cells were also used to evaluate the effects of SMGGT *in vitro*. SMGGT did not have cytotoxicity on HMC-1 cells (Figure 5(a)). SMGGT (10 and 100 *μ*g/ml) reduced the level of VEGF and phosphorylation of ERK in SP/CRH-treated HMC-1 cells, which suggested that the effect of SMGGT might be through suppressing inflammatory response in mast cells (Figures 5(b) and 5(c)).

### 3.6. Quantitative Analysis of the Chemical Constituents of SMGGT

**Figure 6:**
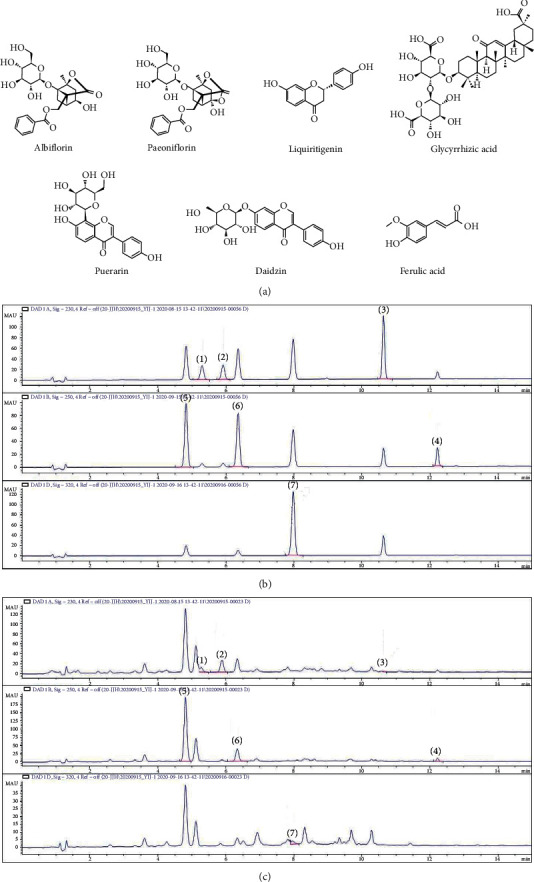
Analysis of chemical constituents of SMGGT by HPLC. (a) Structures of albiflorin, paeoniflorin, liquiritigenin, glycyrrhizic acid, puerarin, daidzin, and ferulic acid. HPLC chromatograms of the standard compounds (b) and SMGGT (c). Albiflorin (1), paeoniflorin (2), liquiritigenin (3), glycyrrhizic acid (4), puerarin (5), daidzin (6), and ferulic acid (7).

Figure 6 shows representative HPLC chromatograms of the SMGGT and the standard compounds. The retention times of puerarin, albiflorin, paeoniflorin, daidzin, ferulic acid, liquiritigenin, and glycyrrhizic acid were 4.829, 5.295, 5.917, 6.369, 7.965, 10.627, and 12.21 min, respectively. The concentrations of these seven components in SMGGT were 0.112–39.843 *μ*g/mg (Table 2).

### 3.7. Network Pharmacological Analysis of SMGGT Compounds and Targets

**Figure 7:**
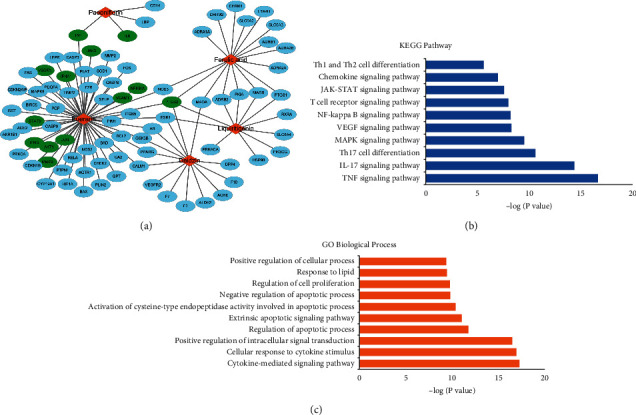
(a) Network of SMGGT compounds and their targets. Diamonds and ellipses represent compounds and targets, respectively. Green ellipses represent AD-related targets. The edges are the compound-target interactions. (b) KEGG enrichment analysis for inflammation-related pathways of 84 targets. (c) GO enrichment for the biological processes of 84 targets.

Network pharmacological analyses were conducted to determine potential targets of SMGGT compounds and their mechanism of action in atopic dermatitis. We identified 84 targets of five compounds of SMGGT (paeoniflorin, puerarin, liquiritigenin, daidzin, and ferulic acid) using the TCMSP database (Figure 7(a)). Targets of albiflorin and glycyrrhizic acid have not been reported in this database. Figure 7(a) shows the relation between SMGGT compounds and their target genes. AD-related targets were indicated according to the DisGeNET database. These genes include AKT1, IFNA1, IFNB, IL-6, JAK3, JUN, MMP-9, NFKBIA, PTGS2, STAT3, TNF, VCAM-1, and VEGFA. Among SMGGT compounds, puerarin possesses the most AD-related targets with 12 targets, while the others possess only target 1-2 AD-related genes. KEGG enrichment analysis was performed to investigate the potential signaling pathways.  Figure 7(b) shows that targets of SMGGT components are involved in several pathways related to inflammatory responses, such as TNF, VEGF, MAPK, NF-kappa B signaling pathways, or AD-specific pathways such as Th1 and Th2 differentiation. Top 10 GO Biological Process terms are shown in Figure 7(c), including cytokine-mediated signaling pathway, cellular response to cytokine stimulus, and positive regulation of intracellular signal transduction, which are the biological processes strongly related to AD.

### 3.8. Docking Analysis of SMGGT Compounds and Targets

**Figure 8:**
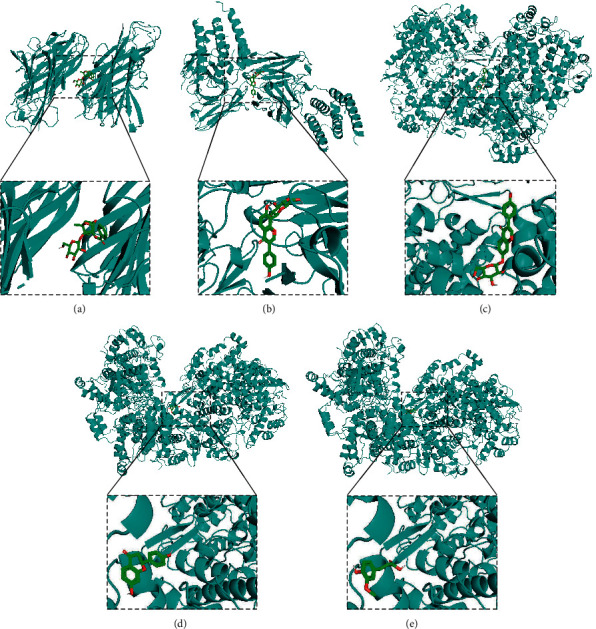
Docking analysis of SMGGT compounds and their target proteins. (a) Paeoniflorin–TNF-*α*. (b) Puerarin–IFN-*α*. (c) Daidzin–COX-2. (d) Liquiritigenin–COX-2. (e) Ferulic acid–COX-2.

We conducted the molecular docking analysis of SMGGT compounds and their 13 AD-related targets. The docking scores indicating affinity between compounds and proteins are shown in Table 3. Most of the ligand-protein binding affinities are less than −6.0 kcal/mol, demonstrating potential binding between SMGGT compounds and targets.  Figure 8 shows the binding modes of representative compounds and targets, including paeoniflorin–TNF-*α*, puerarin–IFN-*α*, daidzin–COX-2, liquiritigenin–COX-2, and ferulic acid–COX-2.

### 3.9. Anti-Inflammatory Effects of Puerarin and Paeoniflorin *In Vitro*

**Figure 9:**
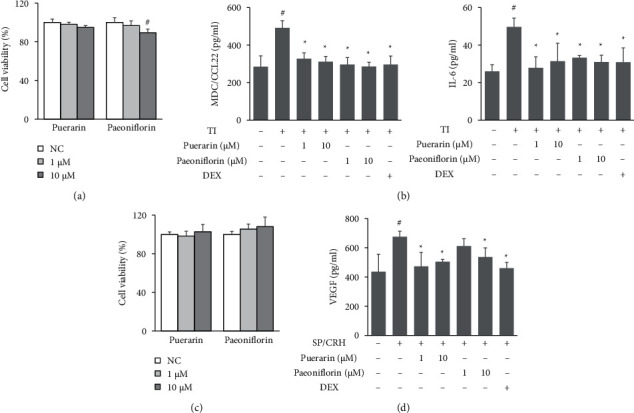
Anti-inflammatory effects of puerarin and paeoniflorin *in vitro*. (a) The viability of HaCaT cells was evaluated using XTT assays. (b) MDC/CCL22 and IL-6 levels in cell culture supernatants. (c) The viability of HMC-1 cells was evaluated using XTT assays. (d) VEGF level in cell culture supernatants. Data are presented as the means ± SDs (*n* = 3 per experiment). ^#^*P* < 0.05 vs. untreated control cells (NC); ^*∗*^*P* < 0.05 vs. TI-treated cells; ^*∗*^*P* < 0.05 vs. SP/CRH-treated cells.

TI-treated HaCaT cells and SP/CRH-treated HMC-1 cells were used to examine the anti-inflammatory effects of two main components of SMGGT, puerarin and paeoniflorin. Pretreatment with puerarin and paeoniflorin significantly reduced the production of MDC/CCL22 and IL-6 in TI-stimulated HaCaT cells as well as decreased VEGF production in SP/CRH-stimulated HMC-1 cells (Figures 9(b) and 9(d)). Puerarin and paeoniflorin did not show any cytotoxic effects in HaCaT and HMC-1 cells (Figure 9(a) and 9(c)).

## 4. Discussion

SD can exacerbate the severity of AD by causing immune dysregulation and inducing psychological stress, leading to significant impairment in the quality of life of patients with AD [1]. In this study, treadmill movement was utilized for the induction of SD in mice. A previous study demonstrated that running on a treadmill can disturb normal sleep homeostasis by reducing time spent in the rapid eye movement (REM) stage, non-rapid eye movement (NREM) stage, and total sleep in the mouse model [28]. Treadmill-induced SD can worsen the AD symptoms in NC/Nga mice by altering the expression of clock genes, in comparison with non-treadmill-treated mice [18]. In the current study, SD exacerbated skin inflammation and triggered behavior changes in DNCB-induced AD mice. SD increased the production of Th2 cytokines (IL-4 and IL-13) significantly, compared to normal mice. These results are in consonance with a previous study that SD favored a shift of the Th1/Th2 balance toward Th2 dominance [29]. Moreover, SD increased the immobility time in the TST in the AD mice.

SMGGT reduced the expression of CD3, a cell surface marker of T cells, and decreased the levels of Th2 cytokines IL-4 and IL-13 in skin lesions in DNCB-treated mice under SD conditions. IL-4 and IL-13 are critical for the production of IgE, recruitment of immune cells, and skin barrier disruption in AD [30]. In addition to its inhibitory effects, SMGGT also reduced the serum levels of IgE and the number of mast cells infiltrated in skin lesions. The chemokines, MDC and TARC, which are expressed strongly in the activated keratinocytes, play a crucial role in the recruitment of Th2 lymphocytes in atopic lesions [31, 32]. *In vitro* results suggested that SMGGT could suppress MDC/CCL22 and TARC/CCL17 production by suppressing p38 and STAT1 phosphorylation and the nuclear translocation of NF-*κ*B in HaCaT cells. These results suggest that SMGGT could ameliorate the SD-worsened AD through anti-inflammatory activity. Interestingly, topical dexamethasone reduced inflammatory cytokines and cellular infiltrates in skin lesions, but they were unsuccessful in alleviating gross skin symptoms. This could be because, despite their anti-inflammatory qualities, topical corticosteroids interfere with epidermal barrier repair by affecting the formation of stratum corneum lipids [33].

Previous studies reported that the HPA axis is activated by SD [7, 34], which is associated with the upregulation of Th2 cytokines important in the pathogenesis of AD, such as IL-4 and IL-5 [35]. In this study, SD increased the serum levels of the hormones of the HPA axis, including CRH, ACTH, and CORT. CRH exerts its proinflammatory effects by binding to the CRH receptors (CRHR) on mast cells to enhance the production of VEGF; hence, AD can be aggravated [36]. The number of mast cells and VEGF levels in skin lesions from DNCB-treated mice under SD conditions were significantly higher than the only DNCB-treated mice. Treatment with SMGGT reduced the CRH levels as well as mast cell infiltration and skin level of VEGF in SD-exposed AD mice. Our previous study indicated that costimulation of CRH and SP significantly increased the production of VEGF from mast cells *in vitro* [21]. In this study, SMGGT reduced VEGF production significantly by suppressing the phosphorylation of the ERK signaling pathway in SP/CRH-treated HMC-1 cells. The neuropeptide SP has been reported to induce sleep disturbances and itch behavior mediated by mast cells in mice [37, 38]. *In vitro* studies suggested that SP induces the expression of CRHR1 on mast cells and a combination of SP and CRH could enhance the inflammatory responses in mast cells [39]. Overall, the effects of SMGGT on SD-worsened AD symptoms might be mediated by the regulation of CRH and inflammatory responses in mast cells.

SD was reported to cause depression-like behavior in a mouse model [40]. The current study showed that SD increased the immobility time in the TST in AD mice, which was reduced by the SMGGT treatment. Melatonin, a hormone secreted by the pineal gland, plays a crucial role in the regulation of sleep and circadian rhythm [41]. Melatonin also has an antidepressant effect and alleviates AD‐like symptoms by reducing levels of IgE in serum and IL‐4 and IFN‐*γ* production [42, 43]. In this study, under SD conditions, AD mice showed lower melatonin levels than normal mice. Moreover, melatonin levels had negative correlations with the levels of inflammatory cytokines VEGF and IL-4 in skin lesions and the immobility time in the TST; hence, the decrease in melatonin production might occur concomitantly with the increase in the inflammatory response and depression-like behavior in AD mice exposed to SD. These results indicated that the SMGGT treatment recovered the reduction of melatonin level, suggesting that SMGGT might exert its effects on SD-exacerbated AD symptoms and behavior changes by increasing melatonin production.

HPLC analysis showed that puerarin and paeoniflorin are the major components in SMGGT with the highest amounts. Puerarin, the main isoflavonoid derived from the root of *Pueraria montana* var. *lobata* (Willd.) Sanjappa & Pradeep, has been reported to suppress the production of inflammatory mediators in AD models [44]. Paeoniflorin, an active ingredient of *Paeonia lactiflora* Pallas, has therapeutic effects on insomnia by promoting non-rapid eye movement sleep and reducing the wakefulness time in a mouse neuropathic pain model [45]. Moreover, both puerarin and paeoniflorin have antidepressant effects on chronic stress-induced depression in a rat model [46, 47]. In addition, network pharmacological and molecular docking analysis also demonstrated that puerarin and paeoniflorin might exert their effects by targeting AD-related targets, pathways, and biological processes. Puerarin and paeoniflorin also exerted anti-inflammatory effects by reducing the production of inflammatory chemokines and cytokines in HaCaT and HMC-1 cells. These results suggest that puerarin and paeoniflorin may play a role in the regulation of skin inflammation and depression-like behavior in AD under SD conditions.

There are several limitations to our study. First, the extraction method utilized in clinical application and the extraction method employed in this animal experiment are different, and accurate dose conversion between animals and human is challenging. In clinical applications, SMGGT is utilized as a water extract; however, in this animal trial, it was employed as a 30% ethanol extract. Second, the higher dose of SMGGT more effectively alleviated the immobility time and serum CRH and CORT levels than the low dose but not in skin symptoms and inflammatory cytokines level. Hence, further research on the safe and effective concentration of SMGGT in terms of bioavailability and tissue distribution is needed. Third, it is required to investigate whether the increase in serum melatonin caused by SMGGT treatment is due to improved sleep quality and circadian rhythms.

## 5. Conclusion

SMGGT ameliorates SD-exacerbated cutaneous inflammation and depression-like behavior in an AD mice model. This study suggests that SMGGT with puerarin and paeoniflorin as main bioactive components is a promising candidate for treatment in patients with AD under SD conditions.

## Figures and Tables

**Table 1:** Composition of SMGGT.

Puerariae Radix	*Pueraria montana* var. *lobata* (Willd.) Sanjappa & Pradeep
Glycyrrhizae Radix	*Glycyrrhiza uralensis* Fisch.
Cimicifugae Rhizoma	*Actaea heracleifolia* (Kom.) J. Compton
Paeoniae Radix	*Paeonia lactiflora* Pall.

**Table 2:** Quantification of chemical composition in the SMGGT by HPLC.

7.096	*Paeonia lactiflora* Pall.
17.857	
0.112	*Glycyrrhiza uralensis* Fisch.
7.603	
39.843	*Pueraria montana* var. *lobata* (Willd.) Sanjappa & Pradeep
9.743	
0.365	*Actaea heracleifolia* (Kom.) J. Compton

**Table 3:** The docking analysis of SMGGT compounds with 13 AD-related targets.

Target		Docking score (kcal/mol)				
Gene	Protein (PDB ID)	Puerarin	Paeoniflorin	Liquiritigenin	Daidzin	Ferulic acid
*AKT1*	AKT1 (3096)	−7.9				
*IFNA1*	IFN-*α* (3UX9)	−8.9				
*IFNB*	IFN-*β* (1AU1)	−7.7				
*IL6*	IL-6 (4O9H)		−7.2			
*JAK3*	JAK3 (5TTS)	−7.7				
*JUN*	JUN (1JUN)	−5.8				
*MMP9*	MMP-9 (6ESM)	−7.5				
*NFKBLA*	I*κ*B-*α* (6Y1J)	−7.9				
*PTGS2*	COX-2 (3MQE)	−7.2		−7.3	−7.6	−5.4
*STAT3*	STAT3 (6NJS)	−7.9				
*TNF*	TNF-*α* (2AZ5)	−8.8	−8.1			
*VCAM1*	VCAM-1 (1VSC)	−8.1				
*VEGFA*	VEGFA (3QTK)	−8.1				

## Data Availability

The data used to support the findings of this study are included within the article.

## Abbreviations

IL: Interleukin
CCL: C-C motif chemokine ligand
STAT1: Signal transducer and activator of transcription 1
ERK: Extracellular signal-regulated kinase.
